# Program Director and Nephrology Fellow’s Perceptions of Home Hemodialysis Education in the United States

**DOI:** 10.34067/KID.0000000644

**Published:** 2024-11-19

**Authors:** Nupur Gupta, Andrew J. Howard, Christina M. Yuan

**Affiliations:** 1 Division of Nephrology, Indiana University School of Medicine, Indianapolis, Indiana; 2 Nephrology Service, Department of Medicine, Walter Reed National Military Medical Center, Bethesda, Maryland

**Keywords:** daily hemodialysis, hemodialysis, medical education

## Abstract

**Key Points:**

- Our survey reports the existence of home hemodialysis (HHD) curricula, including didactic, outside HHD courses, shared decision-making training, and continuity clinics.
- Fellows attending outpatient clinics were more likely to be confident in their ability and prepared to manage HHD patients.
- The critical barrier to HHD education identified by program directors and fellows was insufficient patients.

**Background:**

Public policy focuses on increasing the prevalence of home dialysis. Home hemodialysis (HHD) education and comfort with the procedure are significant barriers to increasing prevalence. This study examines nephrology fellowship didactic curriculum, training program infrastructure, and barriers identified by both program directors and trainees.

**Methods:**

An anonymous, online survey was developed to assess HHD curriculum in US nephrology fellowship programs. During academic year 2023–2024, all US nephrology program directors (*n*=150) were surveyed and asked to forward survey link to their fellows and to indicate the number to whom they forwarded the link.

**Results:**

Fifty-five (55/150; 37%) US nephrology program directors responded to the survey; 80% completed it. Thirty-seven (37/55, 67%) forwarded the link to their fellows. Fellow response rate was 53/237 (22%); 50/53 completed it (94%). Seventy-five percent of the program directors reported either having an HHD curriculum or were developing one. Program directors reported that didactic lectures (87%) were the most frequently available curriculum component, whereas fellows report education on counseling (72%) was most frequent. Sixty percent of fellows and 86% of program directors reported fellow attendance at HHD longitudinal/continuity clinic (routinely or as part of a block rotation). Both peritoneal dialyses and fellows identified insufficient patients as a key barrier to implementing HHD curriculum. Fellows who attended outpatient HHD clinic felt more confident and prepared in HHD-related competencies.

**Conclusions:**

The HHD curriculum exists as didactic lectures, attendance at outside HHD courses, and ESKD-shared decision-making at training programs. Most programs also have continuity clinics. Our findings highlight the presence of curriculum although inconsistent. Fellows who worked in clinic were more likely to be confident and more prepared to manage HHD patients. In addition, fellows with longitudinal clinic felt better prepared than those attending block rotations. Training programs should consider incorporating HHD longitudinal clinical rotations, although this may require creativity to achieve.

## Visual Abstract

**Figure:**
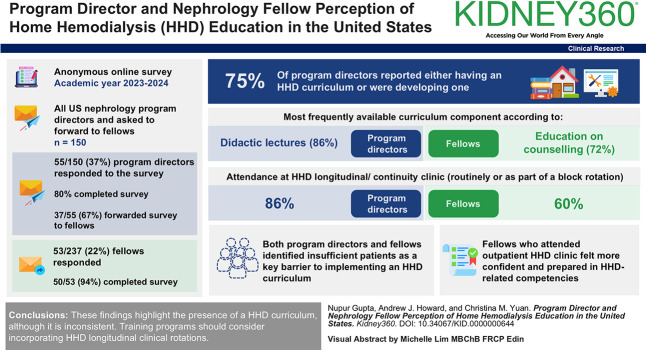

## Introduction

The 2019 Advancing American Kidney Health Initiative set a goal for ≥80% of new patients with ESKD to receive home dialysis or kidney transplant by 2025.^1,2^ Home dialysis modalities include peritoneal dialysis (PD) and home hemodialysis (HHD). Policy proposals for increasing home dialysis prevalence focus on payment models (ESKD Treatment Choice), administered by the US Centers for Medicare & Medicaid Services, to incentivize physicians and dialysis facilities to encourage greater use of home dialysis and kidney transplants. These incentive payment models began in January 2021.^3^ The 2023 United States Renal Data System reported that rates of PD and kidney transplantation have increased, but HHD prevalence has remained quite low (approximately 13,000 patients in 2021 versus 67,000 on PD and 470,000 on in-center hemodialysis).^4^

One barrier, among many, to increasing HHD prevalence may be a lack of familiarity with the modality among nephrologists and the lack of sufficient training during nephrology fellowship. Gupta *et al.*^5^ surveyed 110 nephrology trainees at a home dialysis conference to determine their level of confidence in managing HHD and found that it was low, with 67% reporting that they were not prepared or minimally prepared to manage HHD after graduation. However, this study was limited by a small number of participants and potential bias in that the participants were trainees at a home dialysis conference. Didactic curriculum, training program infrastructure, and clinical rotations were not addressed.

The need to prioritize HHD is especially relevant now because the Accreditation Council for Graduate Medical Education (ACGME) program requirements for Nephrology (2024–2025) includes a requirement for fellows to demonstrate competence in dialysis therapies, including HHD.^6^ Nephrology program directors may face significant barriers in meeting this program requirement. Therefore, we surveyed nephrology program directors and fellows with the goal of better understanding the design and infrastructure of HHD clinical rotations, didactic curriculum, and perceived barriers to training, including lack of faculty expertise and lack of patient material. We also aimed to assess fellow-reported confidence and preparation level on the basis of clinical HHD experience.

## Methods

The anonymous online program director and fellow surveys (Supplemental Material) were developed to assess the training and educational patterns in HHD at US nephrology fellowship programs. The questions covered fellowship experiences across a wide spectrum of HHD patient care, core knowledge, and procedures, and were, in part, on the basis of the previous survey of Gupta *et al*.^5^ Fellow surveys were parallel to those of the program directors to allow comparisons.

The ACGME public list of 2023–2024 US Nephrology Program Directors was used to identify and contact all program directors by email (*N*=150), inviting their participation. The survey was open between December 4, 2023, and February 4, 2024. Survey links were delivered by email with delivery receipt. Respondent internet protocol addresses were blocked. Reminders were sent at 2-week intervals. Respondents were asked to confirm nephrology program director status. If not, they were directed to the end of survey and asked to forward the link to the program director. Program directors entering the survey were asked to forward the anonymous fellow survey link to their fellows and indicate the number of fellows to whom it was sent. Because there are no public lists of active nephrology fellows, this allowed a known number to be contacted and the response rate calculated. All fellows were potentially represented for a given program, and only fellows from programs with responding program directors were included. Program directors had no access to fellow responses, and program and fellow responses were not linked. We have used this method successfully in previous surveys.^7^ A study flow diagram is shown in the Supplemental Material.

Surveys were preceded by an explanatory text, allowing potential respondents to disengage if they did not wish to participate. Demographic information was limited to program size, geographic census area, years in practice (program directors), and fellowship year (fellows).

The protocol (WRNMMC-EDO-2023-1111, 966508) was approved by the Walter Reed National Military Medical Center Department of Research Protections as exempt from Institutional Review Board review per 32 CFR 219.104(d)(4)(ii).

### Statistical Analysis

The response rate was calculated as complete/partially complete surveys divided by number contacted (*N*=150). The response rate for fellows was calculated as the number of partial or complete responses divided by the total number reported as having been sent the link by the program directors. The analysis was descriptive, with percentages, medians, and ranges used as appropriate. Comparison of individual respondents/participating program demographics (program size and geographic location) to the entire population surveyed (to assess nonresponse bias), and comparison of parallel question responses between program directors and fellows were performed using the *t* test, chi-square, or Fisher exact tests. *P* < 0.05 was considered significant. Where *P* < 0.05, effect sizes are expressed as 95% confidence interval for the *t* test and Cramer’s V for chi-square and Fisher exact tests.^8^

## Results

The US nephrology program director's response rate was 37% (55/150), with an average completion time of 6 minutes and 80% completion rate (see Supplemental Material for question-by-question completion). Thirty-seven program directors (25% of programs) forwarded the survey to 237 fellows. The response rate for fellows was 22% (53/237), and 94% (50/53) completed the survey (see Supplemental Material for question-by-question completion). Among the fellows who responded, 47% were first-year fellows (25/53) and 53% second-year fellows (28/53). Program, fellows, and program director demographics are shown in Table 1, with comparisons with programs nationally.^9^ The geographic distribution of programs and the number of clinical fellows per program did not differ significantly from that nationally, although the response rate was higher from program directors in the south.

**Table 1:** Demographics, response, and completion rates of nephrology program directors and fellows

Demographics	Program Directors	Fellows	Programs Nationally (*N*=150)
Number surveyed	150	237	NA
Response, No. (%)	55 (37)	53 (22)	NA
Forwarded surveys to fellows, No. (%)	37 (25)	NA	NA
ACGME-approved program size (number of fellows)^a^	7±3 (*N*=43)	7±4 (*n*=34)	7±3 (*N*=150)
Survey completion, %	80	94	NA
**Year of training, %**			
First-year fellow	N/A	47	NA
Second-year fellow	N/A	53	NA
**Geographic census area^b^, No. (%)**			
Northeast	13 (28)	14 (26)	50 (33)
South	18 (39)	16 (30)	53 (35)
Midwest	9 (20)	11 (21)	28 (19)
West	6 (13)	12 (23)	19 (13)

ACGME, Accreditation Council for Graduate Medical Education; NA, not applicable.

a Mean number of ACGME-approved fellow positions reported by peritoneal dialysis respondents versus mean number of fellows nationally; *P* = 0.86, and by peritoneal dialysis who forwarded the survey link to their fellows (*N*=34); *P* = 0.64 (unpaired *t* test).

b Respondent geographic distribution (%) is compared with geographic distribution of programs nationally. Peritoneal dialysis: *P* = 0.92; fellows: *P* = 0.29 (chi-square).

### HHD Curriculum

Seventy-five percent (34/45) of program directors reported having either an HHD curriculum or were expanding an existing one; 18% (8/45) did not have a curriculum but were developing one; and 7% (3/45) had no curriculum. As shown in Table 2, didactic lectures, outside HHD courses, and ESKD shared decision-making/counseling were the four most frequent curriculum components described by the program directors, and the fellow survey corroborated this. Interestingly, programs with no existing curriculum (*n*=3) and those in the process of developing one (*n*=8) reported having curriculum components similar to those with an existing curriculum. Only 3/50 (6%) fellows reported no exposure to HHD curriculum. Of the 11 program directors who as yet had no curriculum, 7/11 (64%) offered didactic lectures; 8/11 (73%) taught shared decision-making/counseling; and 8/11 (73%) sent fellows to outside HHD courses.

**Table 2:** HHD curriculum components reported by nephrology program directors and fellows^a^

HHD Curriculum Components	Program Directors (*n*=45)	Fellows (*n*=50)
Didactic lectures	87% (39)^a^	60% (30)^a^
Didactic problem sets	18% (8)	26% (13)
Teaching HHD counseling for patients with ESKD	69% (31)^a^	72% (36)^a^
Referral of patients to an ESKD education program that includes information about HHD	78% (35)^a^	52% (26)^a^
Use of home dialysis machines for CRRT	51% (23)	28% (14)
Some or all attend an HHD training course	73% (33)^a^	54% (27)^a^
Dedicated HHD block rotation at primary training site	13% (6)	16% (8)
Dedicated HHD block rotation at associated training site	4% (2)	8% (4)
Attendance at longitudinal HHD continuity clinic	62% (28)	38% (19)
HHD simulations	7% (3)	8% (4)
Referral to unassociated HHD program (patients not followed thereafter)	2% (1)	2% (1)
Nursing staff train interested patients to self-cannulate	29% (13)	20% (10)
Fellows care for in-center nocturnal dialysis patients	9% (4)	12% (6)
No exposure to HHD at the training program	0% (0)	6% (3)

CRRT, continuous renal replacement therapy; HHD, home hemodialysis.

a The four most frequently reported curriculum components reported by program directors and fellows.

### Clinical HHD Rotations

Sixty-seven percent (30/45) of program directors reported availability of an HHD program at the primary training site, while 31% (14/45) reported no HHD program at the primary site but provided an opportunity at different site. Two percent (1/45) reported no program available at any site. The response rate varied question by question among program directors (Supplemental Table 1). Eighty-six percent (37/43) of programs have fellows attending a longitudinal HHD clinic, while 14% of respondents did not have clinic. Of the programs with continuity clinics, 40% (15/37) of programs have monthly continuity clinic; 27% (10/37) have a required block rotation, 10% (4/37) have elective block rotation, and 19% (7/37) reported other experiences.

Only one program had individual fellow patient panels. Overall HHD patient census was >20 in 19% (8/43) of programs, while 28% (12/43) each had 1–5 or 6–10 patients. 86% (32/37) of these programs have 1–5 HHD patients seen by fellows during a continuity clinic. Fellows indicated that 73% (36/49) have oversight by faculty expert in HHD.

The response rate for the fellows to each question varied (Supplemental Table 2). Forty-eight percent (24/50) of fellow respondents attended an HHD longitudinal/continuity outpatient clinic, while 52% (2/40) did not. Among those with continuity clinic, 79% (19/24) typically saw 1–5 patients per clinic session, and 21% (5/24) saw 6–10 patients per session. The structure of continuity clinics varied among programs; 67% (16/24) had either required or elective block rotations, while 25% (6/24) had a monthly clinic, 4% (1/24) weekly clinic, and 4% (1/24) attended four half days yearly.

### Fellow Confidence and Preparedness in Management of HHD Patients

Most were not confident in HHD-related tasks (Table 3). Overall, 22% were not trained in writing HHD prescriptions, followed by water quality management (20%). As shown in Table 3, fellows who attended an HHD outpatient clinic (*n*=24) were more likely to be very confident/confident in management of HHD patient tasks than those who did not attend a continuity clinic (*n*=26; *P* = 0.0001; chi-square=18.4; degrees of freedom 1; Cramer’s V=0.32).

**Table 3:** Fellow self-reported confidence in being able to manage specific HHD tasks

Task	HHD Clinic Experience^a^		All Responding Fellows (*n*=50)
	Sees HHD Patients in Outpatient Clinic (*n*=24)	Does Not See HHD Patients in Outpatient Clinic (*n*=26)	
**Writing/adjusting an HHD prescription, No. (%)**			
Very confident or confident	8 (33)	3 (12)	11(22)
Somewhat confident	3 (13)	5 (19)	8 (16)
Not so confident	12 (50)	7 (27)	19 (38)
Not confident at all	0 (0)	1 (4)	1(2)
Not trained	1 (4)	10 (38)	11(22)
No response	0 (0)	0 (0)	0 (0)
**Vascular access management, No. (%)**			
Very confident or confident	8 (33)	5 (19)	13 (26)
Somewhat confident	13 (54)	7 (27)	20 (40)
Not so confident	0 (0)	4 (15)	4 (8)
Not confident at all	2 (8)	2 (8)	4 (8)
Not trained	1 (4)	6 (23)	7 (14)
No response	0 (0)	2 (8)	2 (4)
**Different types of HHD machines, No. (%)**			
Very confident or confident	11 (45)	2 (8)	13 (26)
Somewhat confident	4 (17)	10 (40)	14 (28)
Not so confident	7 (29)	5 (20)	12 (24)
Not confident at all	0 (0)	2 (8)	2 (4)
Not trained	2 (8)	6 (24)	8 (16)
No response	0 (0)	1 (4)	1 (2)
**Management of water quality in the home, No. (%)**			
Very confident or confident	5 (21)	0 (0)	5 (10)
Somewhat confident	8 (33)	3 (12)	11 (22)
Not so confident	7 (29)	12 (26)	19 (38)
Not confident at all	2 (8)	3 (12)	5 (10)
Not trained	2 (8)	8 (31)	10 (20)
No response	0 (0)	0 (0)	0 (0)

HHD, home hemodialysis.

a Very confident or confident overall in four tasks related to home hemodialysis care: fellows who see home hemodialysis outpatients versus those who do not, *P* = 0.0001 (chi-square=18.4; degrees of freedom 1; Cramer’s V=0.32).

As shown in Table 4, 70% of fellows reported feeling less than fully/moderately prepared to care for HHD patients after graduation; 24% reported feeling not at all prepared. However, fellows who attended an HHD outpatient clinic were significantly more likely to feel some degree of preparedness to care for HHD patients on graduation versus those who did not attend an HHD continuity clinic.

**Table 4:** Self-reported degree of preparation to care for HHD patients after graduation

Outpatient HHD clinics	Fully/Moderately Prepared	Less than Fully/Moderately Prepared	Not Prepared
Sees HHD outpatients (*n*=24)^a^, No. (%)	10 (42)	14 (58)	0 (0)
Does not see HHD outpatients (*n*=26)^a^, No. (%)	5 (19)	9 (35)	12 (46)
All fellow respondents (*n*=50), No. (%)	15 (30)	23 (46)	12 (24)

HHD, home hemodialysis.

a Preparation of fellows seeing HHD outpatients versus those that did not see HHD outpatients. Fisher exact (Freeman-Halton) for 2×3 contingency table. *P* = 0.0003 (chi square=14.7; degrees of freedom 2; Cramer’s V=0.54).

In exploratory analyses, we compared the effect of the number of patients per outpatient session on preparedness and confidence. There was no significant difference in confidence (confident/very confident) for fellows seeing 1–5 patients (*n*=19) versus those seeing 6–10 (*n*=5) (29% versus 60%; *P* = 0.11; Fisher exact test). Moreover, there was no difference in preparedness (fully to moderately prepared): 37% versus 60%; *P* = 0.61; Fisher exact test). In another exploratory analysis, we compared fellow-reported confidence and preparedness in those who saw 1–5 HHD patients/session either monthly or weekly (*n*=7) and those who saw 1–5 per session during a block rotation (*n*=12). There was no difference between the groups regarding preparation (43% versus 33% fully to moderately prepared, *P* = 1.0). However, those who saw patients either monthly or weekly were more confident than those who had experienced a block rotation (46% versus 19%; *P* = 0.017; Cramer's V=0.29).

### Barriers to HHD Training

**Figure 1:**
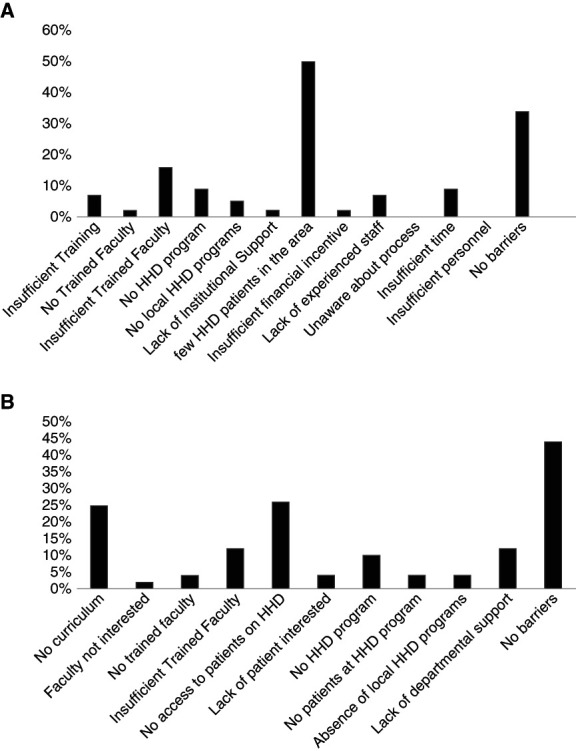
**Perceived barriers to development of home hemodialysis education.** (A) Barriers to effective HHD training perceived by program directors at their fellowship program (*N*=44). (B) Barriers to effective HHD training perceived by fellows at their fellowship program (*N*=50). HHD, home hemodialysis.

The most common program director-cited barrier to incorporating HHD into the curriculum was a lack of HHD patients (22/44). Thirty-four percent (15/44) perceived no barriers (Figure 1A). They also cited the need for more trained faculty (8/44; 18%) and more time to establish a curriculum (7/44; 16%). Only 1/44 (2%) indicated that there were no HHD-trained faculty available. Forty-four percent (22/50) of fellows perceived no barriers, but 26% (13/50) reported insufficient patients, and 25% (12/50) reported a lack of curriculum as barriers to HHD education (Figure 1B).

## Discussion

Our study shows that surveyed nephrology fellowship programs have existing HHD curricula, which included didactic lectures, sending fellows to outside HHD courses, and ESKD-shared decision-making/counseling training. Most programs have HHD continuity clinics, although the frequency of fellow attendance varies and patient numbers are low. The primary barrier identified by PDs and fellows to an effective HHD curriculum was a lack of patients. Fellows with outpatient HHD clinic experience were more likely to be confident in their ability to manage HHD patients and feel more prepared to manage them after graduation. Fellows with a monthly clinic may be more likely to feel confident in their ability to manage HHD-specific tasks than those attending block rotations.

Several studies have reported the need for a structured curriculum for home dialysis. The American Society of Nephrology's Task Force identified a gap regarding home dialysis in current training.^10,11^ Gupta *et al.*ʼs^5^ study in 2021 highlighted the absence of a structured curriculum, especially for HHD. However, our survey reports noteworthy progress in the prevalence of the HHD curriculum in most programs over the last 5 years, although opportunities may be fragmented. Most programs offer opportunities for didactic lectures, outside courses, and counseling training. Clinical exposure to HHD machines, self-cannulation teaching by nurses, and opportunities to attend longitudinal clinics exist. One plausible reason for the lack of consistent curriculum between programs is a lack of guidance from the ACGME. In the nephrology program requirements, HHD was not delineated separately from PD until the 2024–2025 training year. Most of the curriculum milestones are intertwined with in-center HD, which makes it challenging for programs to develop resources.^9^ Our findings urge professional societies, such as the American Society of Nephrology and the National Kidney Foundation, to define and develop HHD curriculum components to help fellowship training programs. Opportunities for programs to collaborate to deliver curriculum and clinical experiences should also be encouraged.

Outpatient clinics are a crucial component in graduate training, significantly impacting fellows’ confidence and preparedness.^12^ Outpatient clinics were commonly available, as described by both fellows and PDs. Fellows who attended outpatient clinics were more confident in managing HHD-specific tasks and more prepared, irrespective of other options. Our study suggests that attendance at a weekly to monthly clinic allows more frequent exposure and builds fellow’s confidence. These clinics allow fellows to participate in patient HHD training and troubleshoot complications. They witness the benefits of HHD, including flexibility of schedules and improved quality of life, which may not be noticeable in a sick patient in the hospital. This underscores the crucial role of outpatient clinics in fellows’ training and their future practice.^13^ In addition, attending outpatient clinics allows fellows to interact with other home dialysis interprofessional team members absent in inpatient settings. Our findings advocate for including HHD outpatient clinics in training, although it may be challenging because HHD clinics are often centralized, located distant from the academic medical center, and may increase fellow travel time. Box 1 discusses the proposed structure of a longitudinal HHD continuity clinic.

Box 1 **Structure of HHD continuity clinic**

Duration—6 mo or more for an academic year

Frequency—half day every month, including additional time allotted to participate in patient training

Educational component

 • Home dialysis counseling

  An opportunity to counsel patients with CKD on options for KRT and especially discuss home hemodialysis (HHD) in detail. Learn to identify barriers and available resources to overcome them

 • HHD training

  ○ Vascular access training techniques, including point of care ultrasound versus models

  ○ Machine knowledge

  ○ Social support both for partner and patients

  ○ Home remodeling if needed

  ○ Dialysis prescription (dry weight, frequency, time)

 • Monthly clinic

  ○ Vascular access health

  ○ Treatment frequency

  ○ Dialysis prescription

  ○ Volume management (ultrafiltration)

  ○ Anemia

  ○ Mineral bone disease

 • Long term complications—cardiovascular disease vascular access complications

Additional opportunities

 • Continuous quality improvement at HHD clinic

 • Rotation with interventional nephrologist/radiologist to understand troubleshooting and anatomy of vascular access

 • Attend at least one quality assessment and performance improvement meeting

Several barriers exist regarding curriculum development for HHD. Program directors believed that insufficient patients were a significant hindrance, but many reported no barriers. Most fellows perceived no barriers, but many also reported that they had no access to HHD patients. The low prevalence and geographic variability of HHD in the United States poses a barrier to adequate clinical experience.^14^ Even in locations with HHD clinics, the number of patients on HHD remains sparse. Residential Van (RV) HHD tours have been used for peer-to-peer mentoring for in-center hemodialysis patients.^15^ HHD patients advocate dialyzing in these vans, which are set up like patient homes. They allow in-center HD patients to experience first-hand setup and treatment outside their outpatient dialysis clinic instead of traveling to a home dialysis clinic. These RV HHD tours could be hosted by academic institutions in partnership with large dialysis organizations for an immersive HHD education experience. The RV could travel to conferences and adjunct learning, like procedure simulations provided in surgical fields. Another alternative is using virtual reality simulation concurrent with hands-on clinical experience. This technology is making an impact on medical education, further use of the metaverse may be able to extend nephrologist and patient training for HHD.^16^ All these alternatives require departmental commitment and resources, which program directors also highlighted as a constraint. As barriers may be, in some cases, unique to each program and region, HHD curriculum is an opportunity for program continuous quality improvement, with evaluation and re-evaluation of the effectiveness of the present HHD curriculum.

This study has limitations. These include the small number of participants and response rates of 22% for fellows and 37% for PDs. Of the 150 programs in the United States, 25% had responses from the program directors who forwarded the survey link to their fellows. This could limit generalizability. However, response rates in this range are not unexpected for an online survey of physicians,^17^ and participants were located geographically and reported program sizes similar to programs nationally. The fellows received the survey link from program directors, and this may have contributed to response bias. Responding program directors may have been more likely to have an HHD curriculum, but it is difficult to predict the direction of bias. Recall bias may also be a problem concerning the clinical experience as would be differences between first-year and second-year fellow clinical experiences. Differences in fellow participation across programs and differing sizes of the programs could also bias the results.

Despite the above limitations, our study has several strengths. We surveyed both program directors and fellows to understand the differences in perception regarding HHD curriculum. Second, the survey examined the existing curriculum and barriers to guide training programs. Third, our study is first to report the curriculum components resulting in the preparedness and confidence of trainees rather than just the perception of program directors and chairs, as previously reported.

Our study concludes that HHD curriculum is currently present in nephrology training programs in the United States. Attendance at longitudinal outpatient clinics seems to be associated with better trainee-reported preparedness and confidence. Inadequate numbers and uneven distribution of HHD patients remain a constraint for adequate training. Considering our findings, training programs must consider incorporating HHD longitudinal clinical rotations, if possible, to optimize fellow preparation to care for HHD patients after graduation.

## Supplementary Material

## Data Availability

All data are included in the manuscript and/or supporting information.

## Supplemental Material

Study Flow Diagram

Nephrology Program Director Survey

Nephrology Fellow Survey

Supplemental Table 1. Program director survey individual question completion.

Supplemental Table 2. Fellow survey individual question completion.

Nephrology Program Director Comments

Nephrology Fellow Comments
