# Supplementary material for: Program Director and Nephrology Fellow’s Perceptions of Home Hemodialysis Education in the United States
Source: Kidney360. 2024 Nov 19;6(2):257–64. doi: 10.34067/KID.0000000644 (PMC11882253; doi:10.34067/KID.0000000644)
Supplement: Supplementary file 1 [file kidney360-6-257-s001.pdf]

ASN American Society of Nephrology
JASN Journal of the American Society of Nephrology
KIDNEY360 Accessing Our World From Every Angle
CJASN Clinical Journal of the American Society of Nephrology

ASN Journal Disclosure Form

As per ASN journal policy, I have disclosed any financial relationships or commitments I have held in the past 36 months as included below. I have listed my Current Employer below to indicate there is a relationship requiring disclosure. If no relationship exists, my Current Employer is not listed.

N. Gupta reports the following:
Employer: Indiana University; Consultancy: Fresenius Medical Care advisory board; Patents or Royalties: UpToDate; Advisory or Leadership Role: Boomrang venture studios; and Other Interests or Relationships: Member, ASN Quality Committee, Member ASN EPC, TDAT.

I understand that the information above will be published within the journal article, if accepted, and that failure to comply and/or to accurately and completely report the potential financial conflicts of interest could lead to the following: 1) Prior to publication, article rejection, or 2) Post-publication, sanctions ranging from, but not limited to, issuing a correction, reporting the inaccurate information to the authors' institution, banning authors from submitting work to ASN journals for varying lengths of time, and/or retraction of the published work.

Name: Nupur Gupta
Manuscript ID: K360-2024-000581R1
Manuscript Title: Program director and nephrology fellow perception of Home Hemodialysis education in United States.
Date of Completion: September 26, 2024
Disclosure Updated Date: September 26, 2024

ASN American Society of Nephrology
JASN Journal of the American Society of Nephrology
KIDNEY360 Accessing Our World From Every Angle
CJASN Clinical Journal of the American Society of Nephrology

ASN Journal Disclosure Form

As per ASN journal policy, I have disclosed any financial relationships or commitments I have held in the past 36 months as included below. I have listed my Current Employer below to indicate there is a relationship requiring disclosure. If no relationship exists, my Current Employer is not listed.

A. Howard reports the following:
Employer: Walter Reed National Military Medical Center

I understand that the information above will be published within the journal article, if accepted, and that failure to comply and/or to accurately and completely report the potential financial conflicts of interest could lead to the following: 1) Prior to publication, article rejection, or 2) Post-publication, sanctions ranging from, but not limited to, issuing a correction, reporting the inaccurate information to the authors' institution, banning authors from submitting work to ASN journals for varying lengths of time, and/or retraction of the published work.

Name: Andrew J. Howard
Manuscript ID: K360-2024-000581R1
Manuscript Title: Program director and nephrology fellow perception of Home Hemodialysis education in United States
Date of Completion: September 25, 2024
Disclosure Updated Date: September 25, 2024

ASN
American Society of Nephrology
JASN
Journal of the American Society of Nephrology
KIDNEY360
Accessing Our World From Every Angle
CJASN
Clinical Journal of the American Society of Nephrology

ASN Journal Disclosure Form

As per ASN journal policy, I have disclosed any financial relationships or commitments I have held in the past 36 months as included below. I have listed my Current Employer below to indicate there is a relationship requiring disclosure. If no relationship exists, my Current Employer is not listed.

C. Yuan reports the following:
Employer: Walter Reed National Military Medical Center; and Other Interests or Relationships: Administrator and Member, Nephrology Education Research and Development Consortium (https://nerdc.org).

I understand that the information above will be published within the journal article, if accepted, and that failure to comply and/or to accurately and completely report the potential financial conflicts of interest could lead to the following: 1) Prior to publication, article rejection, or 2) Post-publication, sanctions ranging from, but not limited to, issuing a correction, reporting the inaccurate information to the authors' institution, banning authors from submitting work to ASN journals for varying lengths of time, and/or retraction of the published work.

Name: Christina M. Yuan
Manuscript ID: K360-2024-000581R1
Manuscript Title: Program director and nephrology fellow perception of Home Hemodialysis education in United States
Date of Completion: September 24, 2024
Disclosure Updated Date: April 22, 2024
