# Supplementary material for: Program Director and Nephrology Fellow’s Perceptions of Home Hemodialysis Education in the United States
Source: Kidney360. 2024 Nov 19;6(2):257–64. doi: 10.34067/KID.0000000644 (PMC11882253; doi:10.34067/KID.0000000644)
Supplement: Supplementary file 2 [file kidney360-6-257-s002.pdf]

Supplemental Material

Gupta N, Howard A, Yuan CM. Nephrology Program Director and Fellow Home Hemodialysis Curriculum Survey

Study Flow Diagram	Page 2
Nephrology Program Director Survey	Page 3
Nephrology Fellow Survey	Page 10
Table 1. Program Director Survey Individual Question Completion	Page 16
Table 2. Fellow Survey Individual Question Completion	Page 17
Nephrology Program Director Comments	Page 18
Nephrology Fellow Comments	Page 20

Home Hemodialysis Program Director and Fellow Survey Flow Diagram

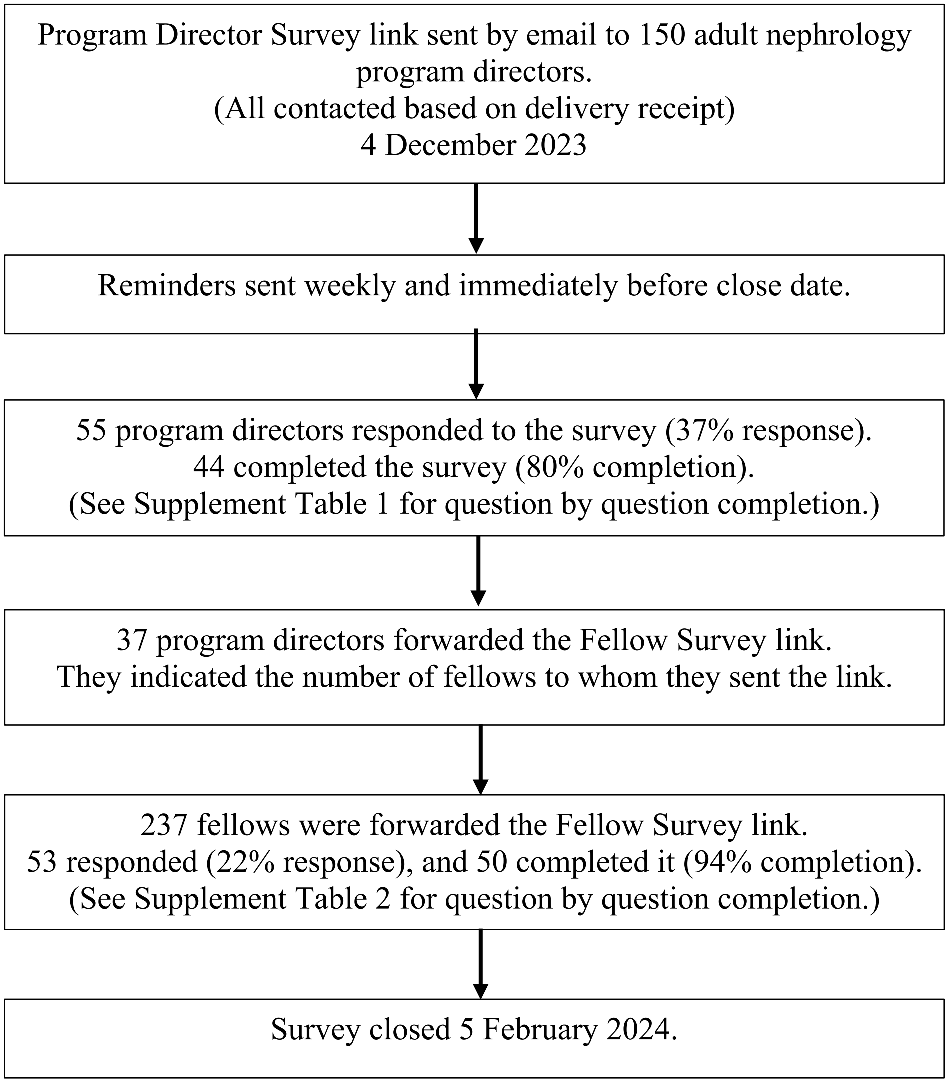

Home Hemodialysis (HHD) Survey for Program Directors

About the Home Hemodialysis Survey

You received this research survey link because you were identified as an adult nephrology program director from the ACGME public list for 2023-2024. If you are not the program director, please do not complete the survey--and if possible, forward the link to the program director. Thank you!

* 1. I am the Nephrology fellowship program director (adult nephrology) at my institution?

- ◯ Yes
- ◯ No

Home Hemodialysis (HHD) Survey for Program Directors

Survey Description and Forwarding the HHD Fellow Survey

This anonymous on-line research survey is designed to assess how home hemodialysis (HHD) training is being provided to nephrology fellows at training programs in the United States. The proposed ACGME program requirements for nephrology for TY 2024-25 require that graduating fellows "demonstrate competence in ... home hemodialysis". Not only do we want input from program directors, we want fellows' input as well. To do this anonymously, we are asking that you forward/email the survey link below to your fellows. This will allow us to establish how many received the link, and calculate their response rate. The survey link is below:

Survey Link: https://www.surveymonkey.com/r/LRBQMHG

This research survey was reviewed and approved by a Walter Reed National Military Medical Center Exempt Determinations Official. Taking the survey implies that you consent to participating. Thank you!

* 2. I have forwarded the link to my fellows.

- ◯ Yes
- ◯ No

Home Hemodialysis (HHD) Survey for Program Directors

Forwarding the Survey to Your Fellows

* 3. The number of fellows to whom I forwarded the link is:

[ ]

Home Hemodialysis (HHD) Survey for Program Directors

About your program

4. How many total clinical fellow positions (first + second year together) are ACGME-approved for your program for 2023-2024?

[ ]

5. What is the geographic location of your training program?

- ◯ Northeast (ME, NH, VT, MA, NY, CT, RI, PA, NJ)
- ◯ South (MD, DC, VA, WV, DE, NC, SC, GA, FL, AL, MS, TN, KY, LA, AR, OK, TX, Puerto Rico)
- ◯ Midwest (OH, MI, IN, IL, WI, MN, IA, MO, NE, ND, SD, KS)
- ◯ West (CA, WA, OR, NV, ID, MT, UT, AZ, NM, CO, WY, HI, AK)

6. The number of years I have practiced nephrology (including fellowship training) is

- ◯ < 5 years
- ◯ 5-10 years
- ◯ > 10-20 years
- ◯ > 20 years

7. The patients served by our training program are predominantly

- ◯ urban
- ◯ suburban
- ◯ rural
- ◯ Other (please specify)

[ ]

Home Hemodialysis (HHD) Survey for Program Directors

About your HHD Curriculum

Definitions regarding possible components of the HHD curriculum:
-An "Outside HHD Course" is a training course (either on-site or virtual) that is offered to fellows and other nephrology providers that covers Home Dialysis (and may also include peritoneal dialysis training in addition to HHD training).
-"Block Rotation" is defined as a rotation lasting longer than 1 week.
-"Continuity Clinic/Longitudinal Clinic" is defined as a clinic in which HHD patients are seen repeatedly (usually monthly) for routine management.

8. Do you have a HHD curriculum?

- ◯ Yes.
- ◯ Yes, and I am planning to expand it.
- ◯ No, but I am developing one.
- ◯ No.

9. Please check all that apply from the list below regarding the home hemodialysis curriculum at your fellowship program:

- ☐ Didactic lecture(s) on HHD during the fellowship.
- ☐ Didactic problem sets for managing HHD orders during the fellowship.
- ☐ In clinic, we ensure fellows properly counsel patients who have CKD5 to consider HHD for kidney replacement therapy.
- ☐ Fellows refer patients to a ESKD education program for shared decision-making that includes information about HHD.
- ☐ Fellows are familiarized with home dialysis machines by using them for CRRT.
- ☐ Some or all of our fellows attend an outside HHD course.
- ☐ We have a dedicated HHD block rotation (longer than 1 week) at our institution/primary training site home dialysis unit.
- ☐ We have a dedicated HHD block rotation (longer than 1 week) at a secondary associated site's home dialysis unit.
- ☐ Fellows attend a HHD longitudinal/continuity clinic (primary or associated site)
- ☐ We use HHD simulations to train fellows.
- ☐ We refer interested patients to a local unassociated HHD training program, but do not follow patients thereafter.
- ☐ Our nursing staff train in-center hemodialysis patients to self-cannulate, if the patient is interested.
- ☐ We have an in-center nocturnal dialysis program.
- ☐ We do not have any exposure to home hemodialysis within our training program.
- ☐ Other (please specify)

Screenshot

Home Hemodialysis (HHD) Survey for Program Directors

Exposure of your fellows to patients on HHD

* 10. We have a HHD program/unit at our institution (primary training site).

- ◯ Yes
- ◯ No, but our fellows gain HHD experience at the HHD program/unit at another site
- ◯ No

Home Hemodialysis (HHD) Survey for Program Directors

Description of the HHD program/unit at which your fellows train

11. Please indicate the number of patients presently followed at the HHD program/unit where your fellows train (either your primary or secondary training site).

- ◯ 1-5
- ◯ 6-10
- ◯ 11-15
- ◯ 16-20
- ◯ > 20
- ◯ None

12. Do some or all of your fellows attend a HHD longitudinal/continuity clinic?

- ◯ Yes (either routinely or as part of a block rotation)
- ◯ No

Home Hemodialysis (HHD) Survey for Program Directors

Experience of your fellows in HHD longitudinal/continuity clinic

13. How many patients are typically seen by the fellow(s) during a HHD longitudinal/continuity clinic session?

- ◯ 1-5
- ◯ 6-10
- ◯ >10

14. How frequently do your fellows attend HHD longitudinal/continuity clinic?

- ◯ Approximately Weekly
- ◯ Approximately Monthly
- ◯ As part of a required block rotation (please indicate details in the comments)
- ◯ As part of an elective block rotation (please indicate details in the comments)
- ◯ Fellows are assigned HHD patients and follow them longitudinally for 3 months or more (please indicate details in comments)
- ◯ Other (please describe in the comments)

Comments?

Barriers to Establishing a HHD Curriculum

15. Do you have faculty who are able/competent to manage patients on HHD?

- ◯ Yes
- ◯ No
- ◯ Other (please specify)

16. What are the barriers to incorporating HHD into your curriculum? (Please check all that apply)

- ☐ I have not received sufficient training.
- ☐ I do not have any sufficiently trained faculty.
- ☐ I do not have a sufficient number of trained faculty.
- ☐ We do not have a HHD program at our institution
- ☐ Our institutional leadership feel there is not sufficient financial incentive to establish a HHD program at our institution
- ☐ Lack of departmental or institutional leadership support for developing a HHD program
- ☐ There are no local HHD programs with whom we can associate with as a training site.
- ☐ There are very few HHD patients in our area.
- ☐ We would like to start a HHD program at our institution, but cannot find experienced nursing staff to train patients and administer the program
- ☐ We do not know how to go about the process of establishing a HHD program
- ☐ I do not have sufficient time to establish a HHD curriculum
- ☐ Our department does not have sufficient personnel to establish a HHD program
- ☐ No barriers.

Other (please specify)

17. Please select the 3 most important barriers to incorporating home hemodialysis into your curriculum?

- [ ] I have not received sufficient training.
- [ ] I do not have any sufficiently trained faculty.
- [ ] I do not have a sufficient number of trained faculty.
- [ ] We do not have a HHD program at our institution
- [ ] Our institutional leadership feel there is not sufficient financial incentive to establish a HHD program at our institution
- [ ] Lack of departmental or institutional leadership support for developing a HHD program
- [ ] There are no local HHD programs with whom we can associate with as a training site.
- [ ] There are very few HHD patients in our area.
- [ ] We would like to start a HHD program at our institution, but cannot find experienced nursing staff to train patients and administer the program
- [ ] We do not know how to go about the process of establishing a HHD program
- [ ] I do not have sufficient time to establish a HHD curriculum
- [ ] Our department does not have sufficient personnel to establish a HHD program
- [ ] No barriers.

Other (please specify)

18. Thank you for taking the survey. Do you have any comments?

Home Hemodialysis (HHD) Survey for Nephrology Fellows

About the Home Hemodialysis Survey

This is an anonymous (IP addresses are blocked), 13-question on-line research survey about fellowship training in home hemodialysis (HHD) in the United States. It should take about 5 minutes to complete.

The proposed 2024-2025 ACGME Nephrology requirements include the stipulation that graduating fellows "demonstrate competence in … home hemodialysis". We hope to determine what Nephrology fellows are experiencing in terms of a HHD curriculum, how it varies between programs, what their direct HHD patient experiences are, and whether they feel confident in their ability to manage HHD patient care issues. Your program director has forwarded you the survey link, but will not know whether you complete it, or what your answers are if you do.

This research study was reviewed and approved by a Walter Reed National Military Medical Center Exempt Determinations Official. Taking the survey implies that you consent to participating.

* 1. I am:

- ◯ A first year nephrology fellow
- ◯ A second year nephrology fellow (or more)
- ◯ Not a nephrology fellow

Home Hemodialysis (HHD) Survey for Nephrology Fellows

About your program

2. What is the geographic location of your training program?

- ◯ Northeast (ME, NH, VT, MA, NY, CT, RI, PA, NJ)
- ◯ South (MD, DC, VA, WV, DE, NC, SC, GA, FL, AL, MS, TN, KY, LA, AR, OK, TX, Puerto Rico)
- ◯ Midwest (OH, MI, IN, IL, WI, MN, IA, MO, NE, ND, SD, KS)
- ◯ West (CA, WA, OR, NV, ID, MT, UT, AZ, NM, CO, WY, HI, AK)

Home Hemodialysis (HHD) Survey for Nephrology Fellows

About your Fellowship HHD Curriculum

Definitions regarding possible components of the HHD curriculum:
-An "Outside HHD Course" is a training course (either on-site or virtual) that is offered to fellows and other nephrology providers that covers Home Dialysis (and may also include peritoneal dialysis training in addition to HHD training).
-"Block Rotation" is defined as a rotation lasting longer than 1 week.
-"Continuity Clinic/Longitudinal Clinic" is defined as a clinic in which HHD patients are seen repeatedly (usually monthly) for routine management.

3. Does your program provide training (of any kind) on the topic of HHD?

- ◯ Yes.
- ◯ No.

4. Have you directly cared for HHD patient(s) during your fellowship?

- ◯ Yes
- ◯ Yes, but only while they are inpatients
- ◯ No

5. Please check all that apply from the list below regarding the home hemodialysis curricu
at your fellowship program:

- ☐ Didactic lecture(s) on HHD during the fellowship.
- ☐ Didactic problem sets for managing HHD orders during the fellowship.
- ☐ Teaching on counseling CKD patients about HHD as one of the options for kidney replacement therapy.
- ☐ We refer patients to a ESKD education program for shared decision-making that includes information abou HHD.
- ☐ We become familiar with home dialysis machines by using them for CRRT.
- ☐ Some or all of us attend an outside HHD course.
- ☐ We have a dedicated HHD block rotation (longer than 1 week) at our institution/primary training site home dialysis unit.
- ☐ We have a dedicated HHD block rotation (longer than 1 week) at a secondary associated site's home dialysis unit.
- ☐ Fellows attend a HHD longitudinal/continuity clinic (primary or associated site)
- ☐ We train on HHD using simulations.
- ☐ We refer interested patients to a local unassociated HHD training program, but do not follow patients thereafter.
- ☐ Our nursing staff train in-center hemodialysis patients to self-cannulate, if the patient is interested.
- ☐ Fellows are involved in the care of in-center nocturnal dialysis program.
- ☐ We do not have any exposure to HHD at our training program.
- ☐ Other (please specify)

Home Hemodialysis (HHD) Survey for Nephrology Fellows

Exposure of fellows to patients on HHD

* 6. We have a HHD program/unit at our institution (primary training site).

- ◯ Yes
- ◯ No, but fellows gain HHD experience at the HHD program/unit at another site
- ◯ No

Home Hemodialysis (HHD) Survey for Nephrology Fellows

Description of the HHD program/unit at which you train

7. Do you attend a HHD longitudinal/continuity clinic?

- ◯ Yes (either routinely or as part of a block rotation)
- ◯ No

Home Hemodialysis (HHD) Survey for Nephrology Fellows

Your experience in HHD longitudinal/continuity clinic

8. How many patients do you typically see during a HHD longitudinal/continuity clinic session?

- ◯ 1-5
- ◯ 6-10
- ◯ >10
- ◯ None

9. How frequently do you attend HHD longitudinal/continuity clinic?

- ◯ Approximately Weekly
- ◯ Approximately Monthly
- ◯ As part of a required block rotation (please indicate details in the comments)
- ◯ As part of an elective block rotation (please indicate details in the comments)
- ◯ We are assigned HHD patients and follow them longitudinally for 3 months or more (please indicate details in comments)
- ◯ Other (please describe in the comments)

Comments?

Barriers to Establishing a HHD Curriculum

10. As part of your training, do you have the opportunity to work with faculty who are experts on HHD?

- ◯ Yes
- ◯ No
- ◯ Other (please specify)

[ ]

11. In your opinion, what are the barriers to effective HHD training at your fellowship program? (Please check all that apply)

- ☐ Our program does not have an HHD curriculum.
- ☐ Our training program leaders do not seem to be interested in improving our HHD curriculum.
- ☐ We do not have any trained faculty.
- ☐ We do not have enough trained faculty.
- ☐ We have no access to patients on HHD.
- ☐ The patients I see do not seem to have an interest in training for HHD.
- ☐ We do not have an HHD program at our institution.
- ☐ We have an HHD program, but there are no patients in it.
- ☐ to my knowledge, there are no local HHD units or providers outside of our institution where we could train.
- ☐ There seems to be a lack of departmental or institutional support for a HHD program/unit.
- ☐ No barriers.

Other (please specify)

Home Hemodialysis (HHD) Survey for Nephrology Fellows

Your preparation for managing HHD patients

12. Below are the various subtopics related to HHD. Please indicate your level of confidence dealing with each. If you have not received any training, indicate "Not Trained".

	Very confident	Confident	Somewhat confident	Not so confident	Not confident at all	Not trained
Writing and adjusting a HHD prescription	○	○	○	○	○	○
Vascular access managment	○	○	○	○	○	○
Different types of hemodialysis machines available for home use	○	○	○	○	○	○
Management of water quality in the home	○	○	○	○	○	○

Comment?

13. How prepared do you think you will be to care for HHD patients after graduation?

- ○ Not prepared
- ○ Slightly prepared
- ○ Somewhat prepared
- ○ Moderately prepared
- ○ Fully prepared

Comments?

14. Thank you for taking the survey. Do you have any comments?

Supplement Table 1. Program Director Survey Completion--Number of Respondents Answering Each Question

Questions	Required Answer	Number Responding	% Responding
(Logic) 1. I am the Nephrology fellowship program director (adult nephrology) at my institution?	Yes	55	100%*
(Logic) 2. I have forwarded the link to my fellows	Yes	49/55	89%
(Logic) 3. The number of fellows to whom I forwarded the link is:	Yes	35/37	95%
4. How many total clinical fellow positions (first + second year together) are ACGME-approved for your program for 2023-2024?	No	43	78%
5. What is the geographic location of your training program?	No	46	84%
6. The number of years I have practiced nephrology (including fellowship training) is	No	46	84%
7. The patients served by our training program are predominantly	No	46	84%
8. Do you have a HHD curriculum?	No	45	82%
9. Please check all that apply from the list below regarding the home hemodialysis curriculum at your fellowship program:	No	45	82%
(Logic) 10. We have a HHD program/unit at our institution (primary training site).	Yes	45	82%
(Logic) 11. Please indicate the number of patients presently followed at the HHD program/unit where your fellows train (either your primary or secondary training site).	No	43/44	98%
(Logic) 12. Do some or all of your fellows attend a HHD longitudinal/continuity clinic?	No	43/44	98%
(Logic) 13. How many patients are typically seen by the fellow(s) during a HHD longitudinal/continuity clinic session?	No	37/37	100%
14. How frequently do your fellows attend HHD longitudinal/continuity clinic?	No	37/37	100%
15. Do you have faculty who are able/competent to manage patients on HHD?	No	44	80%
16. What are the barriers to incorporating HHD into your curriculum? (Please check all that apply)	No	44	80%
17. Please select the 3 most important barriers to incorporating home hemodialysis into your curriculum?	No	44	80%

*55 program directors responded (150 were surveyed): 37% response

Supplement Table 2. Fellow Survey Completion--Number of Respondents Answering Each Question

**Questions**	**Required Answer**	**Number Responding**	**% Responding**
(Logic) 1. I am: (A first year nephrology fellow; A second year nephrology fellow (or more); Not a nephrology fellow)	Yes	53	100%*
2. What is the geographic location of your training program?	No	53	100%
3. Does your program provide training (of any kind) on the topic of HHD?	No	50	94%
4. Have you directly cared for HHD patient(s) during your fellowship?	No	50	94%
5. Please check all that apply from the list below regarding the home hemodialysis curriculum at your fellowship program:	No	50	94%
(Logic) 6. We have a HHD program/unit at our institution (primary training site).	Yes	50	94%
(Logic) 7. Do you attend a HHD longitudinal/continuity clinic?	No	40	100%
(Logic) 8. How many patients do you typically see during a HHD longitudinal/continuity clinic session?	No	24	100%
(Logic) 9. How frequently do you attend HHD longitudinal/continuity clinic?	No	24	100%
10. As part of your training, do you have the opportunity to work with faculty who are experts on HHD?	No	49	92%
11. In your opinion, what are the barriers to effective HHD training at your fellowship program? (Please check all that apply)	No	50	94%
12. Below are the various subtopics related to HHD. Please indicate your level of confidence dealing with each. If you have not received any training, indicate "Not Trained".	No	48 to 50	91-94%
13. How prepared do you think you will be to care for HHD patients after graduation?	No	50	94%

237 fellows were forwarded the survey link; 53/237 responded (22% response)

Program Director Comments

General Comments
Much of HHD is about how you market KRT early on, the RN staff executing home therapies (it's a lot of work to do HHD vs. PD and I feel they often dissuade HHD), and some of the faculty don't feel comfortable with HHD.
We have three sites where fellows train in home therapies. The VA is only PD. The main academic site is a combination of PD and HHD but slow to grow HHD population. We also have an outreach program which involves Dialysis Clinics in the community and our faculty are starting to grow the HHD populations there but there may only be 1-2 patients being seen at any one time so we have not incorporated into fellow curriculum yet but as the populations grow we will.
**Barriers to Training and Curriculum**
Difficulties with the dialysis provider we are contracted with
We are growing the population but slow process.
competing program requirements
We have faculty that are trained, a HHD/PD unit, but as stated previously, patients prefer PD - and only 2-3 faculty are truly passionate about HHD (much of this is a sell from the start of discussing KRT options). Additionally, our RNs are too discretionary as to who can and can't do HHD. I find with education, nearly anyone can do it and succeed.
Difficulty in retaining some of our experienced nursing staff, more so since COVID.
Our President, CEO, COO do not support dialysis let alone Home hemodialysis programs despite proposing to them and urging them to do so. Medicare does not reimburse all costs for Home dialysis. Multiple business models have been proposed to our COO and CEO but they all have turned a deaf ear. I hold didactic sessions based off ASN curriculum on Home Hemodialysis for our fellows yearly. I hold orientation lectures in the first year and invite Home dialysis vendors (Baxter and Nx Stage) with their machines to teach our fellows at the start of their fellowship training, for them to learn hands on experience on the machine. Every advanced CKD 4 patient is referred from my clinic to Home dialysis program for a full education on PD as well as Home HD and transplant education under "options for RRT". In addition our fellows are taught about prescription in home hemodialysis, how frequency of home hemodialysis and not only time matters to achieve adequacy based on weekly clearance calculations by Gotch et. al, and how one can modify prescriptions to achieve adequacy. Fellows learn the role of slow dialysate flow rates and its effect on clearance, how to calculate volume of dialysate needed etc. Despite us having a Home Hemodialysis License and dedicated Home hemodialysis RN and a Home Hemodialysis program and trying to grow our own program our own Institutional support and hospital support is totally lacking as they feel it is too costly and a lack of any interest by them. Due to ABIM and ACGME training requirements I have also partnered with a Home dialysis program where fellows rotate one month block in their second year in addition to what I offer to them in our program to learn both PD and Home HD with hands on experience. This is because our volumes in home dialysis program are low in our own program. Each fellow rotates there in addition to spending some days of some weeks at our own Institutional home dialysis program during their "access rotation months" as an additional option. This is done so that our fellows get some continuity clinic experience and see actual training being given to new patients, if they are interested. All fellows are aware of ABIM letter from ASN about minimum number of new PD patients and longitudinal follow up on PD patients required for graduation that go into effect July 1, 2024 semester. There were no guidelines as to the number of patients to be seen by fellows for Home HD by ASN nor by ABIM. Our Program Letter of Agreement has been in place with our collaborating institution for Home dialysis education and training of our fellows for over 5 years now and our block schedule shows that they rotate there. Our own Institution did not want to support the training of our fellows but our

GME program did. Thus the lack of our Institutional support is the driving force to prevent patients going onto to Home dialysis, Our COO and CEO decided not to pay the salary scales home dialysis RN's are requesting and they do not want to pay for many other costs home dialysis patients incur from vendors. I have personally met and have made multiple requests and meetings for over 6 years now with our COO and requests to our CEO, but it has received a deaf ear primarily due to costs as our patient population is not able to afford the additional costs.
HHD training is currently a part of fellows' ambulatory block and is elective. They are only assigned to HHD clinic or training center if they express interest in doing so.
We are growing the population but slow process.
we would like to enhance the experience but there are more and more competing requirements for training resources
As above we have 3 who are trained well in HHD - but they may view this as excessive time commitment in convincing the patient, training, etc.

Fellow Comments

**General Comments**
There should be core requirement of Home Hemo rotation. It should be separated from other rotations.
Most of my hhd knowledge has come from the HDU.
I will recommend to include the HHD training at the different institutions and we need to facilitate and educate the patients more about HHD.
I wish it could be a mandatory training for all of us.
We have more training for home HD starting next year
Our program encourages us to attend outside HHD lectures - however they don't pay for it- it comes out of our CME allowance . We also don't have protected time for it. We have to pay back the days we spend in outside HHD programs. The outside programs are very educational however they are cramped into 2-3 day session and at least for me the knowledge retention rate is poor
I am interested in HHD so I have pursued additional training outside of what is provided in my program. I am hopeful to translate this to my future practice and I am seeking more opportunities for exposure during my training.
I am 7 months away from graduation and I have encountered 1 HHD patient.
**Training and Curriculum**
We train with NxStage machines for CRRT, thus somewhat confident in using these machines, however have not been trained on home use of these machines.
Most HHD training occurring during second year at my program
We would be joining the attendings at the home HD clinics from next year
Low HHD patient volume
2 faculty gave hhd patients but only 1 fellow is exposed to them
I have outpatient dialysis rotation as an elective multiple times and I regularly attend HHD clinic taking care of pts with HHD.
We are assigned 4 half days of HHD clinic yearly
During elective blocks you are expected to spend time at the HHD clinic, outside of initial participation it is left to the learner how much time they want to spend with the HHD clinic vs pursuing other educational goals (transplant, PD clinic, or interventional neph)
